# Evaluation of a Simulation Program for Providing Telenursing Training to Nursing Students: Cohort Study

**DOI:** 10.2196/67804

**Published:** 2025-03-12

**Authors:** Ola Ali-Saleh, Layalleh Massalha, Ofra Halperin

**Affiliations:** 1 Department of Nursing, Max Stern Yezreel Valley College, Emek Yezreel, 193000, Israel, 972 523216544

**Keywords:** simulation-based training program, telenursing, simulation, program, training, nursing student, nursing care, Israel, nurse-patient relationship, telehealth nursing, remote nursing care, undergraduate, cohort study, knowlege; self efficacy; skills; attitudes

## Abstract

**Background:**

Telenursing has become prevalent in providing care to diverse populations experiencing different health conditions both in Israel and globally. The nurse-patient relationship aims to improve the condition of individuals requiring health services.

**Objectives:**

This study aims to evaluate nursing graduates’ skills and knowledge regarding remote nursing care prior to and following a simulation-based telenursing training program in an undergraduate nursing degree.

**Methods:**

A cohort study assessed 114 third-year nursing students using comprehensive evaluation measures of knowledge, skills, attitudes, self-efficacy, and clinical skills regarding remote nursing care. Assessments were conducted at 2 critical time points: prior to and following a structured simulation-based training intervention.

**Results:**

Participant demographics revealed a predominantly female sample (101/114, 88.6%), aged 20‐50 years (mean 25.68, SD 4.59 years), with moderate to advanced computer and internet proficiency. Notably, 91.2% (104/114) had no telenursing exposure, yet 75.4% (86/114) expressed training interest. Statistical analyses demonstrated significant improvements across all measured variables, characterized by moderate to high effect sizes. Key findings included substantial increases in telenursing awareness, knowledge, skills, attitudes and self-efficacy; significant reduction in perceived barriers to remote care delivery; and complex interrelation dynamics between variables. A multivariate analysis revealed nuanced correlations: higher awareness and knowledge were consistently associated with more positive attitudes and increased self-efficacy. Positive attitudes correlated with enhanced self-efficacy and reduced perceived barriers. Change score analyses further indicated that increased awareness and knowledge facilitated more positive attitudinal shifts, while heightened awareness and positive attitudes corresponded with decreased implementation barriers.

**Conclusions:**

The study underscores the critical importance of integrating targeted telenursing training into nursing education. By providing comprehensive preparation, educational programs can equip students to deliver optimal remote care services. The COVID-19 pandemic has definitively demonstrated that remote nursing will be central to future health care delivery, emphasizing the urgent need to prepare nursing students for this emerging health care paradigm.

## Introduction

### Background

In recent decades, telehealth—the use of information and communication technology in health care—has become a global priority [1]. Telenursing, a subset of telehealth defined as “the use of technology to deliver nursing care and conduct nursing practice” [2], has emerged as a significant health care option. Indeed, telenursing enables patients to access health care providers remotely through various technologies, including mobile devices, computers, and videoconferencing [3]. The American Nurses Association defines telenursing as the use of “technology to deliver nursing care and conduct nursing practices” [4].

Telenursing offers numerous benefits, including improved access to care, savings in time and resources, and enhanced self-care opportunities [5]. Telenursing has been found effective in reducing the number of outpatient and emergency room visits, shortening hospital stays, and lowering health care costs [6]. It has also proved beneficial in educating patients, promoting self-care competence, and providing cost-effective mental health support [7], as well as in providing care for chronically ill patients [5], oncology patients [6] and palliative care [8].

The COVID-19 pandemic accelerated the adoption of telenursing by emphasizing its crucial role in disaster and public health emergency responses [9]. This shift highlighted the need for integrating telenursing concepts into nursing education at both undergraduate and graduate levels [3,10].

Despite the growing importance of telenursing, it is often underrepresented in nursing curricula. Poreddi et al [11] found that while nurse interns generally hold positive perceptions of telenursing, their knowledge of the subject is limited. This gap underscores the need to incorporate telenursing concepts into nursing education in order to prepare future health care providers for an increasingly digital environment. Nurses play an indispensable role in telehealth implementation, with their skills and attitudes serving as supportive factors [12].

Nevertheless, telenursing also entails challenges. Indeed, to provide optimal care without physical contact nurses must possess high-level clinical and interpersonal skills [13,14]. A lack of sufficient knowledge and skills constitutes the main obstacle in telenursing [15]. Previous studies have reported that telehealth education in nursing programs is inadequate [16]. To acquire the skills and develop the competencies required for telenursing, students must practice the use of screen technology and virtual access to remote patients, and telenursing should be introduced early in the nursing curriculum [17].

Simulation-based learning has been identified as an effective method for teaching telenursing skills. This approach allows students to practice in a safe and realistic environment in which they can improve their cognitive, emotional, and psychomotor abilities [18,19]. Studies have shown that simulations positively affect self-efficacy, academic motivation, and the acquisition of clinical skills [20,21]. Moreover, experiential education can be used to augment such crucial factors as perceived usefulness, self-efficacy, and innovativeness, thus enhancing our understanding of the effectiveness and implications of telenursing [22].

Glinkowski et al [23] examined telehealth among college nursing students. They found that 67% (207/308) of participants were willing to engage in telehealth services and 69.49% (214/308) agreed that telehealth should be included as part of the nursing education curriculum. Indeed, enhancing understanding of telenursing and establishing a robust human infrastructure among future nursing professionals are of critical importance. Receptiveness and adaptability have emerged as crucial factors in shaping the quality of health care services provided through telenursing [24]. Studies that examined nursing and medical students in the United States and Poland revealed positive perspectives and attitudes toward telehealth and telenursing. Yet, these studies also revealed knowledge gaps among these students, as well as erroneous beliefs regarding the advantages and possibilities of using telenursing in health practice [25-28]. Adequate education is needed to overcome this lack of knowledge and improve students’ attitudes, including more telenursing content [29].

According to Assaye et al [30], the most significant factors influencing perceptions of telenursing among health care providers include technology availability, web access, and lack of telemedicine training. Indeed, nurses with insufficient education and training in the use of technology face difficulties in implementing telenursing [31]. To overcome these difficulties, students must develop a positive and unprejudiced attitude, while acquiring comprehensive knowledge and acknowledging the limitations of these technologies [32].

Despite the importance of integrating telenursing in nursing study programs, in Israel this topic has not been incorporated into the nursing director’s core program and is not taught in practical nursing training programs. Although studies have been conducted on the use of telenursing, very few have examined the issue of training nursing students to use it. Implementing and evaluating such a training program have the potential to help integrate telenursing into the nursing director’s core program.

In conclusion, despite the increasing importance of telenursing in health care delivery, particularly in view of recent global events, its integration into nursing education remains limited, particularly in certain countries, such as Israel. Further research, educational initiatives, and pilot training programs are needed to bridge this gap and ensure that future nurses are adequately prepared for the evolving landscape of health care delivery.

This study evaluates the skills and knowledge of third-year nursing students regarding remote nursing care before and after participation in a simulation-based training program on telenursing as part of their undergraduate nursing degree. To the best of our knowledge, very few studies to date have evaluated programs that use simulation to train nursing students in the provision of nursing care from a distance (telenursing).

### Hypotheses

This study tests 3 hypotheses. The first hypothesis posits that participation in telenursing training will lead to increased awareness, knowledge, and understanding of required skills in telenursing practice. We expect to observe improved attitudes toward telenursing and enhanced self-efficacy, while simultaneously seeing a reduction in perceived barriers to telenursing implementation. The second hypothesis suggests that there will be significant positive correlations between nursing students’ self-efficacy in telenursing and their awareness, knowledge, skill perceptions, and attitudes toward telenursing. Conversely, we anticipate that barriers to telenursing will demonstrate a negative correlation with self-efficacy levels. The third hypothesis proposes that nursing students’ awareness, knowledge, skill perceptions, and attitudes toward telenursing will serve as significant predictors of both initial self-efficacy levels prior to training and the magnitude of change in self-efficacy following the intervention.

## Methods

### Participants

Participants in this study included 114 nursing students in their third year of studies. Most participants were female (101/114, 88.6%), between the ages of 20 and 50 years (mean 25.68, SD 4.59 years), and studying for a first undergraduate degree (107/114, 93.9%) (rather than attempting a career change).

### Instruments

The questionnaire included 6 sections:

1. The Awareness of Telenursing questionnaire included 6 items, scored on a scale of 1-3 (1=know about it, 2=heard about it, and 3=know nothing about it). Sample item: “Telenursing is the most advanced service provided in nursing.” Internal consistency was acceptable in the pretest (α=.70) but low in the posttest (α=.48). A higher mean score reflects higher awareness of telenursing.
2. The Knowledge of Telenursing questionnaire included 10 dichotomous items, scored 0 or 1. Sample item: “Epidemiological patient surveys can be conducted via telenursing.” Low to acceptable internal consistencies were found in the pretest (α=.75) and the posttest (α=.54). A higher summary score reflects a higher level of knowledge of telenursing.
3. The Skills Required for Telenursing questionnaire included 10 items, scored on a scale of 1-5, with 1 indicating a low level and 5 indicating a very high level. Respondents were asked to rate the extent to which they felt that nurses need specific skills for using telenursing. Sample item: “High listening skills and high question asking skills are required for telenursing.” High internal consistencies were found in the pretest (α=.91) and in the posttest (α=.90). A higher mean score reflects a higher level of skills required for telenursing.
4. The Attitudes About Telenursing questionnaire included 13 items, scored on a scale of 1-5, with 1 indicating that the respondent does not agree at all and 5 indicating a very high level of agreement. Sample item: “I believe that telenursing facilitates the provision of equitable service to all patients.” High internal consistencies were found in the pretest (α=.89) and the posttest (α=.89). A higher mean score reflects more positive attitudes about telenursing.
5. The Barriers to Telenursing questionnaire included 9 items, scored on a scale of 1-5, with 1 indicating that the respondent does not agree at all and 5 indicating a very high level of agreement. Sample item: “I did not invest so many years studying just to work in front of a computer. I will miss personal contact with patients and meeting them face to face.” Good internal consistencies were found in the pretest (α=.79) and the posttest (α=.82). A higher mean score reflects a higher level of barriers to using telenursing.
6. The Self-Efficacy in Telenursing questionnaire included 10 items, scored on a scale of 1-5, with 1 indicating that the respondent does not feel certain and 5 indicating that the respondent feels very certain in being able to help the patient follow instructions given over the telephone and understand complex cases presented in that manner. High internal consistencies were found in the pretest (α=.91) and the posttest (α=.95). A higher mean score reflects higher self-efficacy.

### Procedure

This study is a cohort intervention study conducted among all third-year nursing students in the college, which examined level of knowledge, skills and attitudes regarding self-efficacy and clinical skills for telenursing, and willingness to use this method at 2 points in time—prior to and following training.

The training took place in two stages: (1) the students were taught by the course lecturer, who works in this field. Topics of study included diverse nursing practices, ethical aspects of telehealth, clinical skills including communications skills, challenges in telenursing, and tools for coping with complex issues arising from telenursing. (2) Students practiced telenursing through simulations in various nursing areas. During the simulations they practiced treating patients using the telenursing tools and communications skills they had learned and conducted virtual patient assessments and physical examinations.

The participating students answered a questionnaire assessing the research variables prior to and following training.

### Ethical Considerations

The study was approved by the College Ethics Committee, Emek Yezreel Academic College (approval no. YVC EMEK-2023‐87). Students were recruited via the researcher’s research assistant, who asked for volunteers. Before completing the questionnaires, participants were told that participation was voluntary and that they could drop out of the study at any time. They were informed that their opinions were important for constructing the departmental training program and were therefore encouraged to express them. Participants signed informed consent forms prior to participation. All students in the cohort agreed to participate in the study with no compensation provided. The participants’ privacy and identity were protected, and confidentiality was assured in that no identifying information was asked. The study objectives were explained to the participants and the study was conducted according to the academic ethical code.

### Data Analysis

The data were analyzed with SPSS (version 29; IBM Corp). Descriptive statistics were used for the participants’ demographic characteristics and study variables. As the variable of skills required for telenursing (pre and post) was negatively skewed (preskewness −1.74, SE 0.23; postskewness −3.20, SE 0.23), it underwent exponential transformation. Time differences were assessed with 2-tailed paired *t* tests, using Cohen *d* for effect sizes. Change scores were computed as residual gain scores between the pre- and posttests, and Pearson correlations were calculated between the study variables regarding the pretest scores and the change scores. Multiple linear regressions were calculated for self-efficacy in telenursing, using pretest scores and change scores. Awareness, knowledge, skills, attitudes, and barriers to telenursing were defined as predictors.

## Results

### Descriptive Results

Most participants have initially reported a moderate (54/114, 47.4%) or advanced (58/114, 50.9%) level of knowledge in using computers and the web. Most have not been exposed to telenursing (104/114, 91.2%) but were interested in training in it (86/114, 75.4%). Participants’ age was generally not associated with the study variables (*P*=.07 to *P*=.94) and was thus not controlled for. Other demographic variables had low variance. Thus, the first hypothesis was assessed with a series of 2-tailed paired *t* tests. Significant changes were noted in all variables with moderate to high effect sizes (Table 1). Awareness of telenursing, knowledge of telenursing, skills, attitudes, and self-efficacy in telenursing have all significantly increased following participation in the training program, and barriers to telenursing have significantly decreased.

**Table 1.** Means, SDs, *t* values, and Cohen *d* values for the study variables by time (N=114)^a^.

	Pretest, mean (SD)	Posttest, mean (SD)	*t*_113_ (*P* value)	Cohen *d*
Awareness of telenursing	1.75 (0.48)	2.54 (0.36)	15.18 (<.001)	1.85
Knowledge of telenursing	6.84 (2.48)	8.42 (1.55)	6.18 (<.001)	0.76
Skills required for telenursing	4.60 (0.55)	4.77 (0.38)	3.80 (<.001)	0.36
Attitudes about telenursing	3.41 (0.62)	4.06 (0.62)	9.78 (<.001)	1.04
Barriers to telenursing	2.94 (0.61)	2.63 (0.74)	−4.52 (<.001)	0.45
Self-efficacy in telenursing	3.69 (0.79)	4.04 (0.73)	4.88 (<.001)	0.46

a Ranges: awareness of telenursing 1-3; knowledge of telenursing 0-10; and skills, attitudes, barriers for telenursing, and self-efficacy in telenursing 1-5.

Pearson correlations were calculated among the study variables regarding the pretest and change scores. Significant associations were found (Table 2). In the pretest, higher awareness of telenursing, higher knowledge of telenursing, and perception of the higher skills required for telenursing were associated with more positive attitudes and higher self-efficacy regarding telenursing. Furthermore, more positive attitudes about telenursing were associated with higher self-efficacy in telenursing and with lower barriers to it.

**Table 2.** Pearson correlations between the study variables for the pretest scores and the change scores (N=114).

	1	2	3	4	5	6
Pretest						
1. Awareness of telenursing						
*r*	1	0.13	0.12	0.19	–0.13	0.20
*P* value	—^a^	.18	.19	.04	.16	.04
2. Knowledge of telenursing						
*r*	0.13	1	0.15	0.38	–0.06	0.23
*P* value	.18	—	.11	<.001	.53	.02
3. Skills required for telenursing						
*r*	0.12	0.15	1	0.22	0.03	0.19
*P* value	.19	.11	—	.02	.75	.04
4. Attitudes about telenursing						
*r*	0.19	0.38	0.22	1	–0.27	0.20
*P* value	.04	<.001	.02	—	.004	.04
5. Barriers to telenursing						
*r*	–0.13	–0.06	0.03	–0.27	1	0.06
*P* value	.16	.53	.75	.004	—	.51
6. Self efficacy in telenursing						
*r*	0.20	0.23	0.19	0.20	0.06	1
*P* value	.04	.02	.04	.04	.51	—
Change scores						
1. Awareness of telenursing						
*r*	1	0.25	–0.11	0.23	–0.22	0.14
*P* value	—	.006	.26	.01	.02	.14
2. Knowledge of telenursing						
*r*	0.25	1	0.01	0.23	–0.08	0.02
*P* value	.006	—	.95	.01	.42	.85
3. Skills required for telenursing						
*r*	–0.11	0.01	1	0.12	0.01	0.21
*P* value	.26	.95	—	.20	.93	.02
4. Attitudes about telenursing						
*r*	0.23	0.23	0.12	1	–0.38	0.37
*P* value	.01	.01	.20	—	<.001	<.001
5. Barriers to telenursing						
*r*	–0.22	–0.08	0.01	–0.38	1	–0.23
*P* value	.02	.42	.93	<.001	—	.02
6. Self efficacy in telenursing						
*r*	0.14	0.02	0.21	0.37	–0.23	1
*P* value	.14	.85	.02	<.001	.02	—

a Not applicable.

Regarding the change scores, higher awareness of telenursing was associated with higher knowledge of telenursing and both were associated with more positive attitudes regarding it. Furthermore, higher awareness of telenursing and more positive attitudes regarding it were associated with lower barriers to telenursing. Finally, the higher skills required for telenursing, more positive attitudes about it, and lower barriers were associated with higher self-efficacy in telenursing.

### Associations With and Change in Self-Efficacy

Two multiple linear regressions were calculated to evaluate the associations between awareness, knowledge, skills, attitudes and barriers to telenursing, and self-efficacy in telenursing regarding the pretest and the change between the pretest and the posttest. Level of knowledge in using the computer and the web (0: moderate and low, 1: advanced) was entered first, and the study variables or change in the study variables was entered second (Table 3).

**Table 3.** Multiple linear regressions for self-efficacy in telenursing with awareness, knowledge, skills, and attitudes and barriers to telenursing (N=114).

	Pretest scores^a^			Change scores^b^		
	*B* (SE)	β	*P* value	*B* (SE)	β	*P* value
Level of computer knowledge (advanced)	0.36 (0.14)	.23	.01^c^	−0.10 (0.18)	−.05	.58
Awareness of telenursing	0.32 (0.14)	.19	.03^c^	0.03 (0.10)	.03	.73
Knowledge of telenursing	0.07 (0.03)	.22	.02^c^	−0.10 (0.09)	−.10	.27
Skills required for telenursing	0.01 (0.01)	.06	.54	0.19 (0.09)	.19	.04^c^
Attitudes about telenursing	0.21 (0.12)	.17	.09	0.36 (0.10)	.36	<.001^c^
Barriers to telenursing	0.07 (0.11)	.06	.53	−0.09 (0.10)	−.09	.34

a *R*2=0.23, *P*<.001; *F*_6, 107_=5.20, *P*<.001.

b *R*2=0.21, *P*<.001; *F*_6, 107_=4.64, *P*<.001.

c These values are significant.

Both regression models were found significant, with 23% and 21% of the variance in the pretest score and in the change score, respectively, being explained by them. Regarding the pretest score, higher awareness and more knowledge of telenursing were associated with the perception of higher self-efficacy in telenursing. Regarding the change score, greater improvement in the perceived skills required for telenursing and a higher positive change in the attitudes regarding telenursing were associated with a greater improvement in the perception of self-efficacy in telenursing.

## Discussion

This study aimed to examine how a simulation training program on telenursing affected awareness, knowledge, skills, attitudes, self-efficacy, and perceived barriers regarding telenursing among third-year nursing students. The results demonstrate significant improvements across all measured variables, with moderate to high effect sizes, suggesting that the implemented training program was effective. Moreover, the higher skills required for telenursing, more positive attitudes regarding it, and lower barriers were associated with higher self-efficacy in telenursing. These findings emphasize that the simulated experiences served as effective interventions, providing students with innovative learning opportunities [33].

The substantial increase in participants’ awareness and knowledge of telenursing reflects the growing recognition that it is a critical component of modern health care delivery [1,3]. This increase is particularly noteworthy, given that most participants (104/114, 91.2%) had no prior exposure to telenursing, despite their initial moderate to advanced levels of computer and web proficiency. Findings regarding the posttest score of awareness of telenursing and its change score may be biased in unknown ways and should be regarded with caution.

Vaidya [34] recently emphasized the need to offer simulation telehealth education to undergraduate, graduate and health care practitioners in an effort to achieve a more effective remote diagnosis and treatment management for patients in need, such as those living with chronic disease.

The findings of this study are also in line with those of Mun et al [35], which indicated that nursing students lacked substantial awareness regarding telenursing. Nevertheless, the results also portrayed a positive outlook. Indeed, according to Kazawa et al [36], engaging in telenursing helps students enhance their understanding of telehealth practices, develop critical thinking skills, and broaden their knowledge of how to manage and address patient needs in a virtual care setting. Chang et al [37] also found that nurses with telehealth experience have significantly higher perceptions of its usefulness than those with no such experience, and these perceptions correlated positively with attitudes and behavioral intentions. These findings imply that providing nursing students with telenursing education can help them understand and harness this method [28]. Moreover, telenursing education was shown to have a significant impact on nurses’ knowledge, attitudes, and awareness of future work [8,11]. Telehealth simulation was shown to improve nursing students’ professional skills [38].

This study goes a step further by demonstrating that targeted training can significantly improve attitudes. This finding is crucial, as positive attitudes are likely to translate into greater willingness to engage with and implement telenursing practices in future professional roles. Nurses with prior telehealth knowledge had more positive attitudes toward telenursing than those who had never encountered telehealth-related information, and their attitudes toward telenursing correlated positively with their intentions to engage in telehealth [37]. Moreover, nursing students’ attitudes toward telenursing demonstrated a significant correlation with telenursing experience, observation of telenursing during clinical practice, and exposure to telenursing education [35].

The decrease in perceived barriers to telenursing following the training program is a particularly encouraging outcome of this study. It suggests that the program has effectively addressed common concerns and obstacles associated with telenursing implementation and has the potential to offer a smoother integration of these practices in future health care settings.

Indeed, to prepare for their future roles, nursing students need telenursing education [35]. Previous studies examining nursing students reported positive prospects for telenursing, alongside negative perceptions associated with a lack of awareness [28,39]. Furthermore, Mun et al [35] found that nursing students who had a negative outlook regarding telenursing noted its impracticality as compared with face-to-face nursing, as well as the lack of patient contact, challenges faced by older individuals, and accessibility issues for low-income or rural residents. The assumption is that these limited perceptions derive from a lack of formal education in telenursing [40]. This type of education has the potential to enhance knowledge and attitudes regarding telenursing [41]. Indeed, significant improvements in understanding the use and role of telenursing were found among individuals who had undergone telehealth education [42].

This study found that skills and self-efficacy improved following the intervention. These findings are in line with a previous study that found a significant enhancement in skills and self-efficacy following training [37], thus indicating that telenursing education plays a crucial role in improving the type of specialized knowledge required for clinical telenursing. These findings suggest that nursing students need formal education in telenursing. Such education will enhance their competency and nurture a positive attitude, facilitating the seamless integration of telenursing into the digital health care era [35]. Our findings also corroborate those of Reierson et al [17], who emphasized the importance of introducing and practicing telenursing at the beginning of nursing curricula.

Moreover, our study found differences in the predictors of self-efficacy. Prior to the intervention, self-efficacy was a function of awareness and knowledge, whereas following it self-efficacy correlated with a change in skills and attitudes. To the best of our knowledge, almost no intervention studies have been conducted on the role of education in promoting telenursing. Kazawa et al [36] found that telenursing education is essential in expanding nursing students’ knowledge and skills. Moreover, Mun et al [35] found that self-efficacy regarding telenursing among nursing students was associated with telenursing experience, education, and attitudes toward telenursing. Knowledge regarding advance care planning was also found to be associated with self-efficacy [43,44]. Bandura [45,46] also found a relationship between knowledge and skills, which translates into action by increasing self-efficacy to overcome barriers, and Mata et al [47] found that skills can improve health professionals’ performance and self-efficacy.

Former studies of simulation-based instruction in nursing and telehealth were done by Parmeter et al [48], using a posttest-only design. Following peer-to-peer telehealth simulation scenarios via Zoom (Zoom Video Communications, Inc), the students demonstrated a high score of confidence and telehealth performance. These results align with the findings of this study.

These findings have significant implications for nursing education and practice. First, they strongly support the integration of telenursing into nursing curricula, as advocated by Asimakopoulou [3] and Puro and Feyereisen [10]. Moreover, the success of the simulation-based training program in this study is in line with previous research highlighting the effectiveness of simulations in nursing education [20,49]. Simulation can also play an important role in helping students acquire and improve their self-efficacy and nursing skills [50]. Our findings suggest that similar approaches may be valuable in preparing nursing students for the growing prevalence of telenursing in health care delivery.

This study presents 3 key limitations. First, the absence of a control group limits the ability to conclusively attribute observed changes solely to the training program. Future research should incorporate a parallel control group design to enable a more rigorous comparative analysis and establish clearer causal relationships. Second, the focus on immediate posttraining outcomes prevents understanding of long-term telenursing competency retention and clinical application. Implementing a longitudinal study design with multiple follow-up assessments at 3, 6, and 12 months posttraining would provide insights into knowledge and skill sustainability. Third, the study’s recruitment from a single college limits the generalizability of the findings across nursing student populations in Israel. Expanding the research to multiple educational institutions, potentially including diverse geographic and institutional contexts, would enhance the external validity of the results. Cronbach α value for awareness of telenursing was acceptable at pretest (α=0.70) but low at posttest (α=0.48). This finding indicates that the posttest score of awareness of telenursing has a low reliability, and the relevant findings should be regarded with caution. That is, the findings regarding the posttest score of awareness of telenursing and its change score may be biased in unknown ways and should be regarded with caution. Future studies are advised to validate a modified version of this questionnaire or use a different one.

Future research should address these methodological limitations by integrating control groups, longitudinal designs, and multi-institutional sampling to comprehensively evaluate telenursing training programs and their broader implementation potential. In addition, research exploring the implications of telenursing training on patient outcomes and health care system efficiency would provide critical empirical evidence to support broader program implementation.

In conclusion, this study provides compelling evidence for the effectiveness of a simulation-based telenursing training program in enhancing nursing students’ competencies across multiple domains of telenursing. The findings underscore the importance of integrating telenursing education into nursing curricula in an effort to prepare future health care providers for the evolving landscape of health care delivery.

To implement these findings effectively, health care organizations should provide hands-on telenursing training through structured workshops and regular skill refresher seminars for practicing nurses. The Ministry of Health should establish standardized telenursing protocols and mandate their adoption across all health maintenance organizations to ensure consistent quality of care. Medical schools should integrate practical telenursing simulations into their core curriculum, while practicing health care professionals should complete required continuing education modules which include hands-on simulation training to maintain their competency in virtual care delivery.
